# A genetic variant associated with multiple sclerosis inversely affects the expression of CD58 and microRNA-548ac from the same gene

**DOI:** 10.1371/journal.pgen.1007961

**Published:** 2019-02-07

**Authors:** Michael Hecker, Nina Boxberger, Nicole Illner, Brit Fitzner, Ina Schröder, Alexander Winkelmann, Ales Dudesek, Stefanie Meister, Dirk Koczan, Peter Lorenz, Hans-Jürgen Thiesen, Uwe Klaus Zettl

**Affiliations:** 1 University of Rostock, Department of Neurology, Division of Neuroimmunology, Rostock, Germany; 2 Steinbeis Transfer Center for Proteome Analysis, Rostock, Germany; 3 University of Rostock, Institute of Immunology, Rostock, Germany; University of Miami, UNITED STATES

## Abstract

Genome-wide association studies have identified more than 200 genetic variants to be associated with an increased risk of developing multiple sclerosis (MS). Still, little is known about the causal molecular mechanisms that underlie the genetic contribution to disease susceptibility. In this study, we investigated the role of the single-nucleotide polymorphism (SNP) rs1414273, which is located within the microRNA-548ac stem-loop sequence in the first intron of the CD58 gene. We conducted an expression quantitative trait locus (eQTL) analysis based on public RNA-sequencing and microarray data of blood-derived cells of more than 1000 subjects. Additionally, CD58 transcripts and mature hsa-miR-548ac molecules were measured using real-time PCR in peripheral blood samples of 32 MS patients. Cell culture experiments were performed to evaluate the efficiency of Drosha-mediated stem-loop processing dependent on genotype and to determine the target genes of this underexplored microRNA. Across different global populations and data sets, carriers of the MS risk allele showed reduced CD58 mRNA levels but increased hsa-miR-548ac levels. We provide evidence that the SNP rs1414273 might alter Drosha cleavage activity, thereby provoking partial uncoupling of CD58 gene expression and microRNA-548ac production from the shared primary transcript in immune cells. Moreover, the microRNA was found to regulate genes, which participate in inflammatory processes and in controlling the balance of protein folding and degradation. We thus uncovered new regulatory implications of the MS-associated haplotype of the CD58 gene locus, and we remind that paradoxical findings can be encountered in the analysis of eQTLs upon data aggregation. Our study illustrates that a better understanding of RNA processing events might help to establish the functional nature of genetic variants, which predispose to inflammatory and neurological diseases.

## Author summary

More than 200 genetic loci have been associated with an increased risk of developing multiple sclerosis (MS). Here, we investigated the role of a single-nucleotide polymorphism (SNP), which is located within the microRNA-548ac stem-loop sequence in the first intron of the CD58 gene. We analyzed expression data of blood-derived cells of about 1000 subjects and observed that MS risk allele carriers have reduced CD58 mRNA levels but increased hsa-miR-548ac levels. Our findings suggest that Drosha cleavage activity is affected, perhaps attributable to the specific SNP. This may contribute to partial uncoupling of CD58 gene expression and hsa-miR-548ac production from the shared primary transcript in immune cells. We discovered that the mature microRNA downregulates genes involved in inflammatory processes and in controlling the balance of protein folding and degradation. Our study exemplifies that paradoxical findings can be encountered in the analysis of genetic variants regulating transcription and/or RNA processing.

## Introduction

In the past 10 years, genome-wide association studies (GWAS) assaying hundreds of thousands of single-nucleotide polymorphisms (SNPs) rapidly expanded our knowledge of genetic loci contributing to complex multifactorial diseases. The current GWAS catalog contains already more than 89000 unique SNP-trait associations [1], and shared risk variants between diseases are increasingly recognized. However, despite these important insights, timely diagnosis and appropriate treatment of autoimmune diseases remains challenging, because our understanding of pathogenesis is still limited. We have to bear in mind that association does not imply causation. Moreover, onset and course of autoimmune diseases are influenced by a combination of different risk factors, including multiple genetic, epigenetic, immunological, and environmental factors. As in chaotic systems related to the *n*-body problem, all these factors may constantly interact with each other, which provokes unpredictable outcomes [2].

Multiple sclerosis (MS) is a chronic inflammatory disorder of the central nervous system (CNS) and the most common cause of neurological disability in young adults. The disease is characterized by immune cell infiltrates in the CNS, demyelination, and axonal loss [3,4]. Most patients are initially diagnosed with relapsing-remitting MS (RRMS), which lasts for around 2 decades before transition to secondary progressive MS (SPMS), whereas 10–15% of the patients show a gradual worsening of neurological functions from disease onset without early relapses (primary progressive MS, PPMS) [5]. The individual course of the disease is very heterogeneous [6]. Severity and diversity of clinical symptoms largely depend on frequency and distribution of lesions in brain and spinal cord. However, the precise triggers of neuroinflammation and neurodegeneration in MS have not been resolved yet. T-cells have been in the focus of intense research, but evidence is accumulating that B-cells play a crucial role as well. In fact, basically all approved disease-modifying therapies for MS affect the B-cell population [7]. The biological underpinnings of MS are highly complex, involving multifaceted interactions between immune cells as well as between risk genes and environmental factors (e.g., exposure to tobacco smoke and infection with Epstein-Barr virus, EBV) [8,9].

The hereditary component of MS has been first demonstrated in studies of twins. In comparison to the general population, a monozygotic twin of an MS patient has a more than 100 times higher risk to develop the disease [10]. It is well-established that the human leukocyte antigen (HLA) class II region exerts the strongest genetic effect in MS [11]. Beyond that, international GWAS so far identified about 200 independent MS-associated non-HLA variants, each exerting a small effect on disease risk [12–14]. However, still little is known about causal genetic variants and the biological mechanisms that underlie MS susceptibility. Any of the genetic variants within a risk-associated locus that are in strong linkage disequilibrium (LD) with a GWAS-reported SNP could account for the association. Moreover, the vast majority (>90%) of MS-associated tag SNPs are located in intronic or intergenic regions [14]. There is currently a lack of studies aimed at deciphering the functional implications of these loci in MS. It is also unclear to which extent genetic variants may influence the course of MS (relapsing and progressive) and the degree of disease activity.

**Fig 1:**
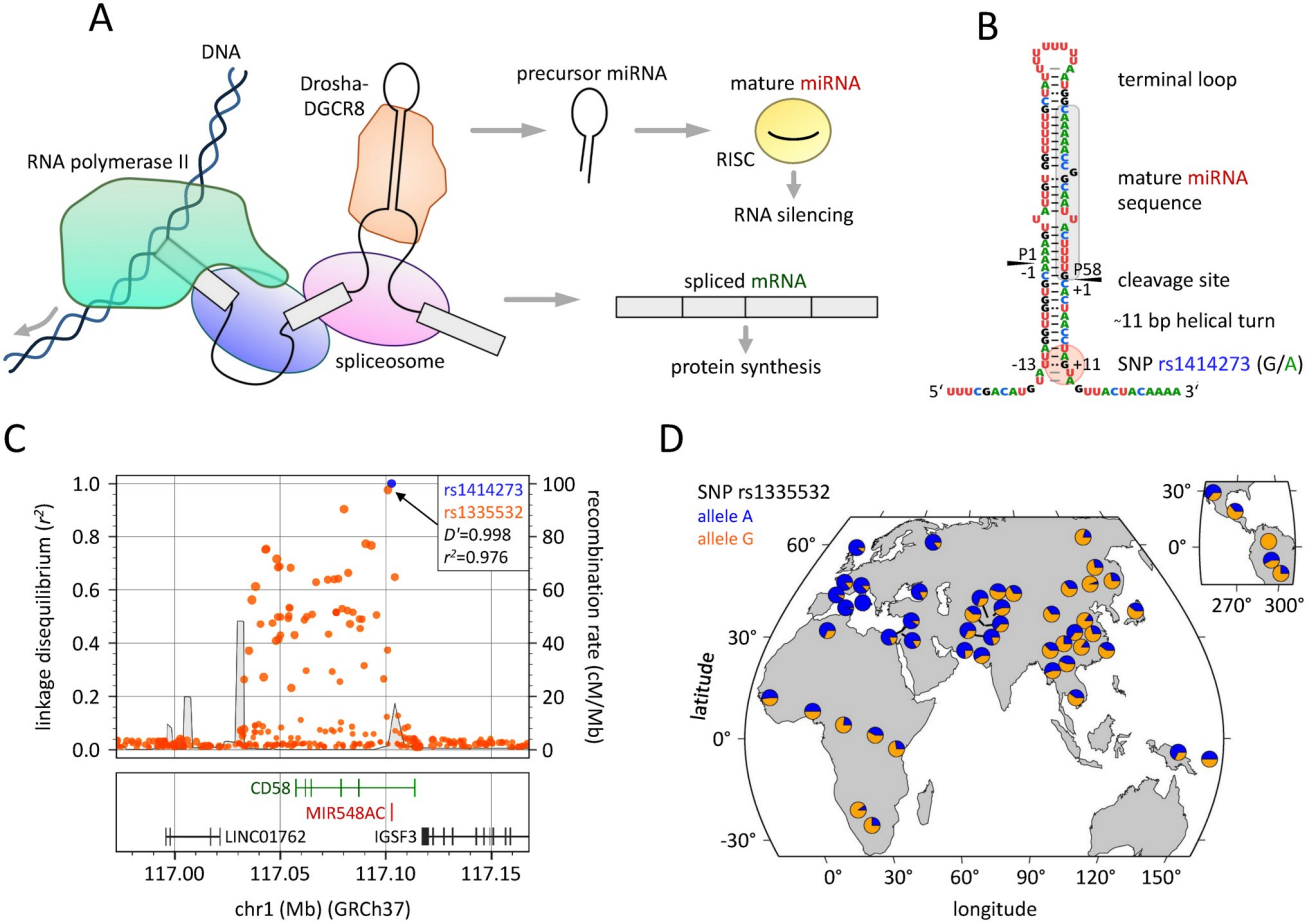
Biogenesis of microRNA-548ac and genetic variants in the CD58 gene locus. (**A**) Diagram of the processing of an mRNA and an intronic miRNA from the same transcript (adapted from [49]). Both RNA splicing by the spliceosome and miRNA stem-loop cropping by the Drosha-DGCR8 complex occur cotranscriptionally. Drosha may cleave the miRNA-harboring intron before splicing commitment of the flanking exons. The resulting precursor miRNA is subsequently processed into a mature miRNA, which is loaded into the RNA-induced silencing complex (RISC). (**B**) Annotated secondary structure of hsa-mir-548ac. Highlighted in gray is the 22 nt long sequence of the mature miRNA isoform as assigned by Jima *et al*. (miRBase accession MIMAT0018938) [43]. The red circle marks the only common single-nucleotide polymorphism (SNP) within the stem-loop region. The G allele is overrepresented in MS patients. (**C**) Genetic variants in pairwise linkage disequilibrium (LD) with SNP rs1414273. This plot was generated using the LDproxy module of the web-based analysis tool LDlink [97]. Shown are *r*^*2*^ LD values of proximal SNPs based on all subpopulations of the 1000 Genomes project (orange dots), recombination rate as estimated from HapMap data (gray line), and the position and exon-intron structure of nearby genes on chromosome 1 (chr1, GRCh37 assembly). The MS-associated SNP rs1335532 is in strong LD with SNP rs1414273 (blue) (correlated forward strand alleles: A = C, G = T). The entire block of LD spans about 50 kb but does not include the promoter region of CD58, which is encoded on the minus strand in the reference genome. (**D**) Worldwide distribution of SNP rs1335532 alleles. Global allele frequencies were visualized as two-color pie charts with the HGDP Selection Browser [98]. The disease susceptibility variant (A, blue) is the major allele in European populations and the minor allele in East Asian and Southern African populations. cM/Mb = centimorgan per megabase, HGDP = Human Genome Diversity Panel, MS = multiple sclerosis.

One of the top non-HLA loci conferring risk of MS is found within the CD58 gene locus [15,16]. In particular, SNPs in the first intron of CD58 (e.g., dbSNP identifiers rs12044852, rs10801908, rs1335532, and rs2300747) are associated with MS, with odds ratios ranging from 1.30 in the latest multi-national GWAS [13,14] to 2.13 in a regional cohort from Germany [16] to 2.63 in familial MS cases [17] as compared to controls. Interestingly, a microRNA (miRNA), hsa-miR-548ac, is encoded in this intron. Thus, both CD58 mRNA and hsa-miR-548ac are processed from the same primary transcript (Fig 1A). The miRNA hsa-miR-548ac belongs to a large family of miRNAs that evolved from a primate-specific transposable element (Made1) [18], and it has been first identified in 2010 by Jima *et al*. based on high-throughput sequencing (HTS) of small RNAs from human B-cells [19]. By post-transcriptional gene silencing, miRNAs are able to modulate the expression of hundreds of genes either directly or indirectly [20,21]. However, the specific target genes of hsa-miR-548ac have so far not been explicitly determined experimentally. It is also important to note that there is a SNP, rs1414273, located in the miRNA stem-loop sequence, 11 nt downstream of the 3' Drosha cleavage site (Fig 1B). This SNP is very commonly inherited together with the MS-associated SNPs from GWAS, with *r*^*2*^ and *D'* as measures of LD being close to 1 (Fig 1C) [16]. Furthermore, the MS risk alleles of these SNPs are the major alleles in individuals of European ancestry but the minor alleles in East Asian populations (Fig 1D). In 2009, De Jager *et al*. could show by an expression quantitative trait loci (eQTL) analysis based on 149 individuals from the HapMap collection that carriers of the MS-associated haplotype express lower levels of CD58 mRNA [15] (as later confirmed at the protein level [22]). The authors also further established the function of CD58 protein in the activation of T-cells through engagement of the CD2 receptor, and they demonstrated that decreased CD58 expression, as particularly seen during a relapse, leads to dysfunction of regulatory T-cells [15]. However, the widespread expression of CD58 in many cell types necessitates caution in the interpretation of the role of CD58 in the pathogenesis of MS. Moreover, the study by De Jager *et al*. did not consider hsa-miR-548ac, which was not known at that time.

We speculated that the MS-associated SNPs within the CD58 gene locus affect the expression of mature hsa-miR-548ac and that, more specifically, SNP rs1414273 is the causal genetic variant that acts as *cis*-mRNA-eQTL and *cis*-miR-eQTL. Here, we provide evidence that the genotype alters the efficiency of the Drosha-DGCR8 complex in processing the miRNA stem-loop. This appears to modulate the expression levels of both hsa-miR-548ac and CD58 mRNA in an inverse and allele-specific manner. We further compared the levels of hsa-miR-548ac in different immune cell subsets circulating in the blood, and we obtained first insights into the function of this miRNA by screening for target genes. In exploring the data, we stumbled on two paradoxes of a three-variable relationship: (1) the non-transitivity paradox of positive correlation and (2) the Simpson's paradox. These are well-known statistical phenomena, especially in the social and biomedical sciences [23,24]. We discovered similar eQTL paradoxes for other human miRNAs. Our study illustrates how disease susceptibility variants may regulate uncoupling of miRNA production from host gene expression. This underscores the contribution of miRNAs in disease pathogeneses and their potential use as molecular biomarkers, which has not been fully exploited yet.

## Results

### Analysis of eQTL in three cohorts

**Fig 2:**
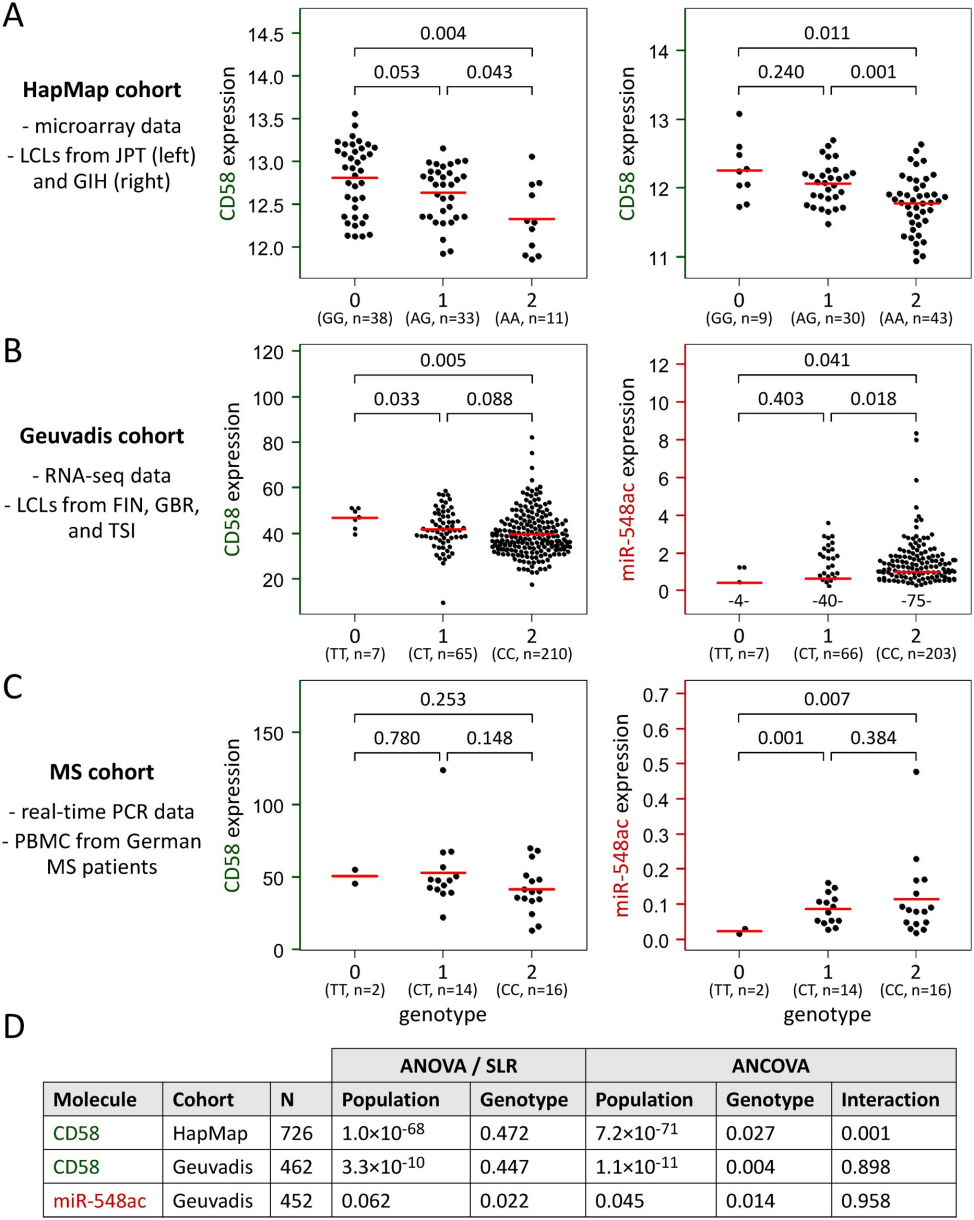
eQTL analysis of CD58 and microRNA-548ac based on three different data sets. Expression values of CD58 mRNA (labeled in green) and hsa-miR-548ac molecules (labeled in red) measured using microarrays (**A**), RNA-sequencing (**B**), and quantitative real-time PCR (**C**) were plotted for each genotype group. Genotypes 0, 1, and 2 denote the number of MS risk alleles carried, defined either by SNP rs1335532 (**A**) or SNP rs1414273 (**B** and **C**). The average expression level per group is indicated by a red line. Welch *t*-test *p*-values are shown above the brackets for all pairwise genotype comparisons. (**A**) HapMap cohort data (in log2 scale) demonstrated a significant relationship between the MS-associated SNP and CD58 transcript levels in independent populations (n = 82 JPT and n = 82 GIH displayed). (**B**) This could be confirmed by Geuvadis cohort data, presented here for LCLs collected from 282 individuals living in Europe. Interestingly, the eQTL effect is in the opposite direction for hsa-miR-548ac: Significantly higher levels of this miRNA were seen in individuals with increased genetic risk of MS. Numbers below the data points specify the proportion of samples (of n = 276 analyzed LCLs) with zero miRNA read counts. (**C**) Data of the regional MS cohort (n = 32) further substantiated the differences in miRNA levels among the genotypes. A non-significant positive correlation of CD58 mRNA and hsa-miR-548ac expression was found in both the RNA-sequencing data (**B**) and the real-time PCR data (**C**). (**D**) The table gives the *F*-test *p*-values calculated for the complete data of the HapMap and Geuvadis cohorts, establishing the *cis*-mRNA-/miR-eQTL when accounting for population structure in the analysis of covariance (ANCOVA). ANOVA = analysis of variance, eQTL = expression quantitative trait locus, FIN = Finnish in Finland, GBR = British in England and Scotland, GIH = Gujarati Indians in Houston, JPT = Japanese in Tokyo, LCL = lymphoblastoid cell line, MS = multiple sclerosis, PBMC = peripheral blood mononuclear cells, PCR = polymerase chain reaction, SLR = simple linear regression, SNP = single-nucleotide polymorphism, TSI = Toscani in Italia.

The eQTL analysis employed microarray data (HapMap cohort), HTS data (Geuvadis cohort), and real-time PCR data (MS cohort). Firstly, we analyzed CD58 mRNA expression in lymphoblastoid cell lines (LCLs) derived from a total of 726 individuals from 8 global populations from the HapMap project [25–27]. The normalized gene expression data correlated significantly with the genotypes of the intronic SNP rs1335532 (used as proxy) for 4 populations: CHB (*F*-test *p* = 0.019), GIH (*p* = 0.00008), JPT (*p* = 0.0004), and MEX (*p* = 0.030). In all these populations, homozygous carriers of the MS risk allele showed, on average, the lowest CD58 transcript levels (Fig 2A). This clearly confirms the eQTL and the protein QTL previously described in LCLs by De Jager *et al*. [15] and Wu *et al*. [22], respectively. The analysis of the other 4 populations did not reach statistical significance, which may be explained by the limited numbers of individuals (n≤135) and distinct risk allele frequencies across the populations (from 0.34 in JPT up to 0.86 in CEU). Moreover, an analysis of variance (ANOVA) revealed huge differences in CD58 gene expression between the 8 populations (*p* = 1.0×10^−68^), impairing the association analysis. In fact, when considering the data of all 726 individuals in a simple linear regression (SLR) model, the eQTL effect could not be seen (*p* = 0.472) due to this confounding. This is reminiscent of Simpson's paradox [23], as elaborated later in this article. The issue of combining different groups of data can be more adequately addressed using an analysis of covariance (ANCOVA), which blends ANOVA and regression. This analysis demonstrated a significant main effect for the rs1335532 genotype (*p* = 0.027) and an interaction between genotype and population (*p* = 0.0007) (Fig 2D).

Next, we evaluated RNA sequencing (RNA-seq) data of the Geuvadis consortium, which provided the levels of CD58 mRNA and hsa-miR-548ac in LCLs derived from 465 individuals from 5 populations [28]. In this cohort, the risk allele frequency of SNP rs1414273 was 0.86 in individuals of European ancestry (CEU, FIN, GBR, and TSI) and 0.40 in YRI. As for the microarray data of the HapMap cohort, significant differences in CD58 expression could be observed in the HTS data between the populations (ANOVA *p* = 3.3×10^−10^). This population effect was modest for hsa-miR-548ac (*p* = 0.062), which was actually detected in only 59.7% of the samples due to limited sequencing depth, with an overall average of 1.2 million miRNA reads per sample after quality control [28]. The eQTL analysis again reflected a Simpson-like paradox: When combining all data, the association of CD58 mRNA expression with the genotype of SNP rs1414273 was not significant in the SLR (*p* = 0.447) but in the ANCOVA (*p* = 0.004), which included the population as independent variable (Fig 2D). The data thus confirm the result of the HapMap cohort analysis, with individuals homozygous for the allele conferring risk of MS having a moderately lower level of CD58 gene transcripts than individuals homozygous for the alternative allele and heterozygous carriers showing an intermediate level of expression. On the other hand, the intronic SNP was also significantly associated with hsa-miR-548ac sequencing counts (*p* = 0.022 and *p* = 0.014 for SLR and ANCOVA, respectively), however, in the opposite direction: The genetic risk variant correlated with higher levels of this miRNA. The pattern of increased miRNA expression and decreased CD58 mRNA expression in carriers of the MS-associated allele was noticed in all 5 populations, but it did not reach statistical significance per population given the limited number of LCLs analyzed (n≤96). In Fig 2B, we visualized the HTS data for non-CEU Europeans (FIN, GBR, and TSI), because they are independent from the LCLs included in the HapMap cohort. In this geographically more proximate subset, the apparent inverse regulatory effect of the rs1414273 polymorphism on levels of CD58 (*F*-test *p* = 0.017) and hsa-miR-548ac (*p* = 0.017 likewise) can be seen.

To verify the findings obtained from the LCL data, we studied peripheral blood mononuclear cells (PBMC) from 32 MS patients from north-east Germany. Using quantitative real-time PCR, we were able to detect mature hsa-miR-548ac molecules in each of the triplicate reactions (threshold cycle C_t_<45). This demonstrates that the measurement sensitivity of the MS cohort analysis is much better than of the HTS-based Geuvadis cohort analysis. Regarding SNP rs1414273, only two MS patients had the TT genotype (with respect to the forward strand of the reference genome). Thus, most patients carried the disease risk variant C at least once (n = 14 CT heterozygotes and n = 16 CC homozygotes). The inverse correlation of the risk allele with lower CD58 mRNA expression and higher hsa-miR-548ac levels, which was seen in the Geuvadis data, could, in principle, be seen in the real-time PCR data as well (Fig 2C). Because of limited sample size and small effect sizes, the genotypic association was not significant for CD58 (*F*-test *p* = 0.172) and hsa-miR-548ac (*p* = 0.158) alone but for the ratio of the levels of miRNA and host gene (*p* = 0.040). Moreover, Welch *t*-tests indicated significantly elevated miRNA expression in patients with the susceptibility variant for MS (Fig 2C). There was no evidence that the data could be otherwise explained by differences in age, gender, therapy, or disease course (*F*-test *p*>0.18).

To conclude, the MS-associated haplotype is implicated with significantly decreased CD58 mRNA levels (HapMap cohort and Geuvadis cohort data). On the other hand, significantly increased levels of hsa-miR-548ac can be seen in risk allele carriers (Geuvadis cohort and MS cohort data). The *cis*-mRNA-/miR-eQTL analysis thus suggests that SNP rs1414273 affects the processing of both mRNA and miRNA from the same primary transcript of the CD58 gene locus.

### Illustration of eQTL paradoxes in simulated data

The eQTL analysis revealed two observations, which are not intuitive. Firstly, different global populations showed similar eQTL results, but the association disappeared when combining the data. Secondly, although transcriptional control of CD58 and hsa-miR-548ac is coupled, there appears to be an inverse regulatory relationship to SNP rs1414273. The latter is presumably driven by a genotype-dependent Drosha-mediated processing of the intronic miRNA stem-loop. As this cleavage occurs cotranscriptionally before splicing catalysis, the mRNA levels of the host gene may be affected as well. Two data sets were simulated for investigating the two paradoxes further.

**Fig 3:**
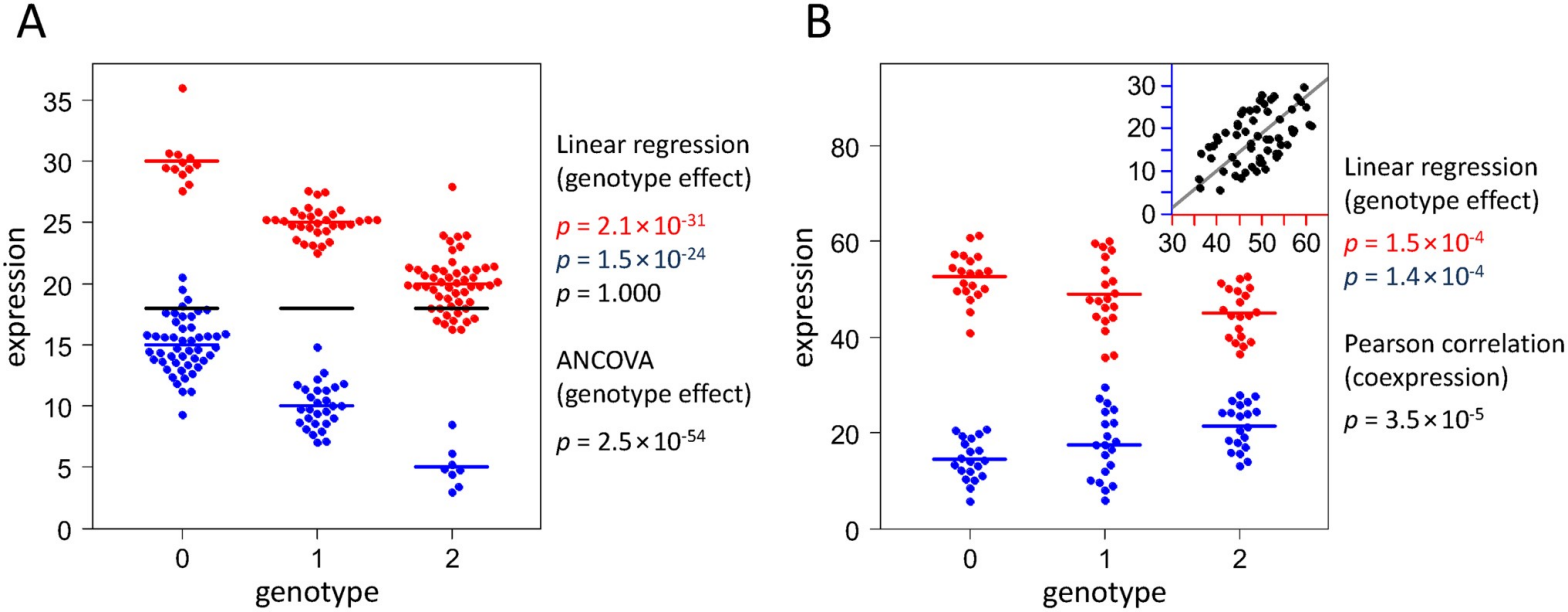
Simulation of paradoxes that may occur in eQTL studies. (**A**) Simpson-like paradox. In this illustrative data set, the genotype of a biallelic single-nucleotide polymorphism (coded in 0-1-2 format) is highly significantly associated with the expression of a gene in two different populations of people (red and blue). However, when combining the data of both populations, this association is completely lost (*F*-test *p* = 1.0). In fact, the average expression level of the whole population (indicated by black horizontal lines) is exactly 18 for each genotype. In this case, a linear model incorporating genotypes as well as populations is useful, because it allows considering the differences in expression between the subpopulations and thus clearly reveals the significance of the eQTL. (**B**) Non-transitivity paradox of correlation. Simulated example of an eQTL shared between two RNAs (red and blue) with opposing directional effects on expression levels. Either allele is associated with increased expression of one RNA and, at the same time, reduced expression of the other RNA. This inverse eQTL effect is not intuitive under the condition that the expression of the two RNAs is significantly positively correlated as visualized in the inset with colored x-axis and y-axis referring to the levels of each RNA and with orthogonal linear regression line shown in gray. ANCOVA = analysis of covariance, eQTL = expression quantitative trait locus.

The Simpson-like paradox is shown in Fig 3A. For both populations examined (n = 96 and n = 84), the eQTL is highly statistically significant (*F*-test *p* = 2.1×10^−31^ and *p* = 1.5×10^−24^). However, when ignoring the subpopulations and looking at the entire cohort (n = 180), the average expression value is exactly 18 for each genotype group. Thus, the genotype effect completely disappeared (*p* = 1.0), because the expression is more strongly influenced by population than by genotype. In a more extreme situation, even a reversal of the genetic association can happen, with the same data leading to opposite conclusions. When incorporating the populations in an ANCOVA, the clear link between SNP and gene expression becomes evident again (*p* = 2.5×10^−54^). This demonstrates that biological or technical factors may mask important effects and that we must be careful when aggregating data.

The second data set refers to the non-transitivity property of correlation (Fig 3B). Here, we investigated a three-way relationship between two RNAs and a SNP (n = 20 individuals per genotype). In these simulated data, individuals with a higher allele count tend to show higher levels of the first RNA (Pearson coefficient *r* = 0.472 and *p* = 0.0001), and the levels of the first RNA and the second RNA are significantly positively correlated as well (*r* = 0.508 and *p* = 0.00003). One might thus assume that a higher allele count is also associated with higher levels of the second RNA. However, this reasoning fails. In fact, the correlation between the SNP allele and the second RNA is negative (*r* = -0.471 and *p* = 0.0001). This seems to be a paradox, but it actually reflects a common misconception concerning transitivity of correlation [24].

The simulations thus provided examples of two statistical phenomena that may occur in eQTL studies. In both cases, there are plausible explanations that can help resolving the paradoxes. Therefore, causal considerations are necessary to avoid contradicting conclusions.

### Genome-wide analysis of eQTLs in microRNA stem-loop regions

We employed the HTS data from the Geuvadis consortium [28] to expose similar *cis*-miR-eQTLs as for hsa-miR-548ac. The data contained the levels of mature miRNAs from a total of 741 miRNA stem-loops and their respective host genes. For 63 of those, we found that the expression of the miRNAs depends on the genotypes of SNPs mapping to the stem-loop sequence or the flanking bases up to positions -25/+25. Nine SNPs were each associated with the production of two mature miRNAs from the same locus—one from the 5' strand and one from the 3' strand of the precursor—either in the same direction or in the opposite direction (e.g., SNP rs2910164 in hsa-mir-146a). For 7 stem-loop regions, 2–3 SNPs were filtered (not just one). Altogether, 80 SNP-miRNA associations were detected by ANCOVA with *p*-value<0.05 for the genotype variable (S1 Table). A subset of 74 associations also showed statistical significance in a linear mixed-effects model (LMM) analysis (S1 Table). In 11 cases, the eQTL effect was not significant when not controlling for the population structure of the data (Simpson-like paradox). In 36 cases, asynchronous correlations between SNP, miRNA, and primary transcript were observed (non-transitivity paradox), and in 27 cases, the miRNA expression was considerably uncoupled from host gene expression (S1 Table). Such uncoupling suggests that local sequence features modulate the activity of RNA processing factors, although the causal events underlying these associations cannot be unequivocally resolved based on this analysis. Different steps in miRNA biogenesis might be independently affected by genetic variants, in particular transcriptional regulation (dependent on LD block length) and RNA cleavage by Drosha (cropping) and Dicer (dicing). Drosha/DGCR8-independent mechanisms also play a role as 7 of the stem-loops are mirtrons, which are processed to precursor miRNAs through splicing, lariat debranching, and trimming [29]. It remains speculative whether the allele-specific expression of some of the identified miRNAs is linked to host gene mRNA destabilization. Several of the gene loci have been reported in GWAS to be affiliated with diseases such as schizophrenia (n = 8) and systemic lupus erythematosus (n = 5) (S1 Table). Intriguingly, apart from hsa-mir-548ac, two more of the determined miR-eQTLs (in hsa-mir-941-4 and hsa-mir-4664) are located within a distance of <200 kb from MS susceptibility tag SNPs [14]. Insights into causal relationships might come into reach by further delineating the genetic effects on the levels of the mature miRNAs.

### Effect of SNP rs1414273 on microRNA-548ac processing *in vitro*

As the eQTL analyses showed that the MS risk locus in the CD58 gene is associated with CD58 mRNA and hsa-miR-548ac expression, we speculated that SNP rs1414273 is the causal variant at this locus. Dependent on the genotype of this SNP, there is either a Watson-Crick base pair (A-U) or a wobble base pair (G-U) at the base of the miRNA stem-loop (Fig 1B) [16]. Cell culture experiments and real-time PCR analyses were conducted to examine whether this difference affects the enzymatic processing of the miRNA.

**Fig 4:**
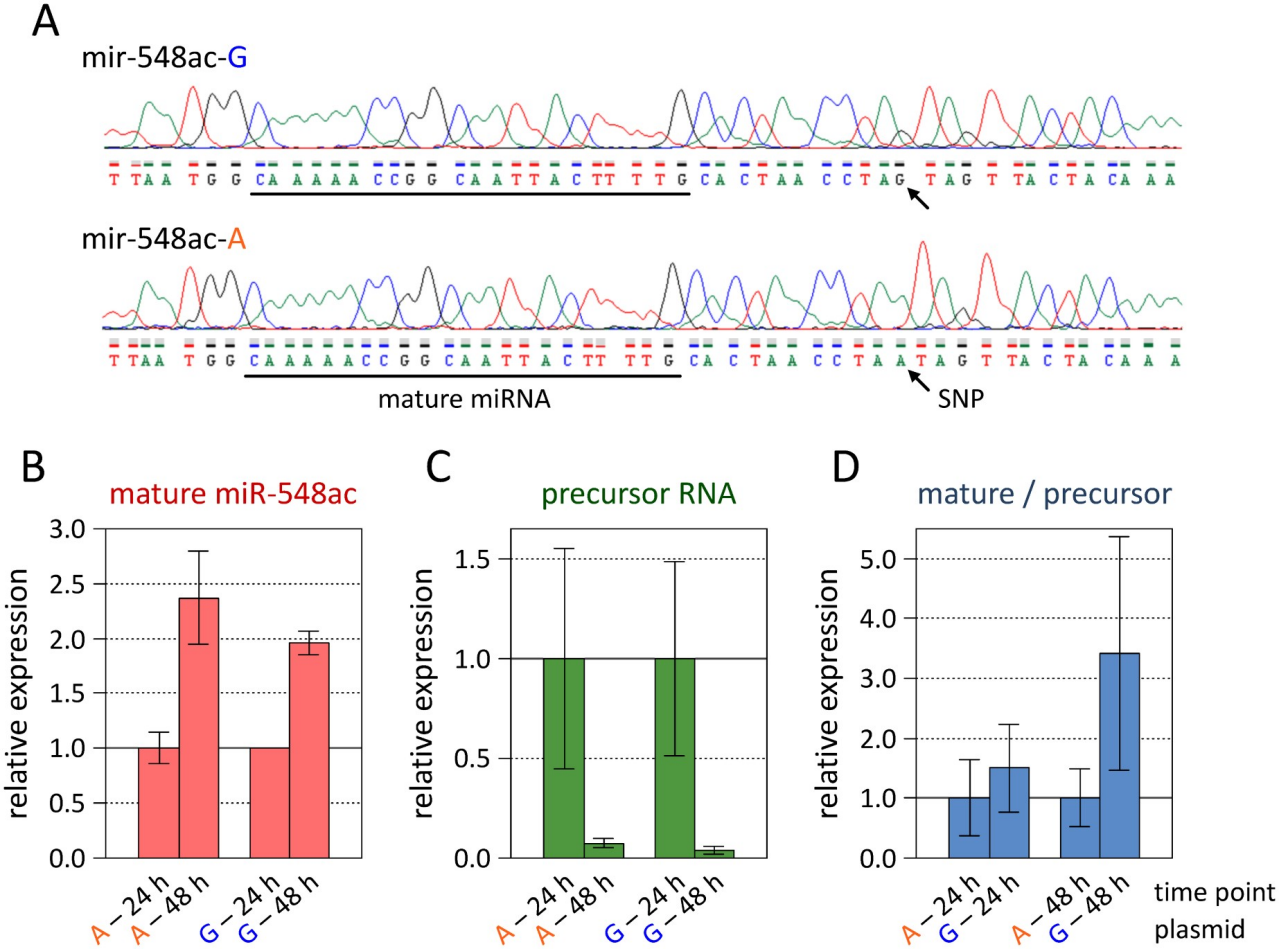
Expression of microRNA-548ac *in vitro* and effect of SNP rs1414273. Precursor miRNA expression plasmids were used to express hsa-miR-548ac in HeLa cells and to examine allele-specific differences. (**A**) Sanger DNA sequencing chromatograms for the sense strand of the original plasmid (mir-548ac-G) and the mutated plasmid (mir-548ac-A), which only differ at a single base 11 nt downstream of the 3' Drosha cleavage site. The two plasmids represent the SNP rs1414273 at the lower stem of the primary miRNA foldback structure. The G allele (in blue) is the MS-associated allele. The sequence of the mature miRNA is underlined. (**B**) Real-time PCR measurement of hsa-miR-548ac molecules that were produced by the endogenous miRNA biogenesis machinery in HeLa cells. The levels of the mature miRNA were roughly twice as high after 48 h than after 24 h post-transfection with either plasmid. (**C**) Relative expression of hsa-mir-548ac precursor transcripts after transient transfection. A more than 10-fold decrease in the precursor levels was consistently observed from the 24 h to the 48 h time point. (**D**) Functional analysis of the possible effect of the genetic variant on hsa-mir-548ac processing efficiency in HeLa cells. At the 48 h time point, a non-significant 3.4-fold higher ratio of mature miRNA levels versus precursor RNA levels was found in mir-548ac-G-transfected cells compared with mir-548ac-A-transfected cells. The PCR data suggest that the A allele might be associated with reduced stem-loop recognition and/or processing by the Drosha-DGCR8 complex. Each bar plot displays the arithmetic means and standard errors of the scaled 2^-ΔCt^ (**B** and **C**) or 2^-ΔΔCt^ (**D**) values. MS = multiple sclerosis, PCR = polymerase chain reaction, SNP = single-nucleotide polymorphism.

After HeLa cells were transiently transfected with precursor miRNA expression vectors carrying either the G allele (mir-548ac-G) or the A allele (mir-548ac-A) (Fig 4A), precursor transcripts as well as mature hsa-miR-548ac molecules could be detected in all PCR wells (n = 24 each), with an average raw C_t_ value of 35.1 and 34.8, respectively. In contrast, both the precursor RNA and the mature miRNA were not detected (C_t_>45 for 23 out of 24 wells) after transfection with a negative control vector with scrambled sequence, which implies that they are not endogenously expressed in HeLa cells. For the mir-548ac-G and the mir-548ac-A plasmids, we observed similar changes in expression over the course of time, with levels of the mature miRNA being increased by 96% and 137% and levels of the primary miRNA transcript being decreased by 96% and 93% at 48 h versus 24 h post-transfection, respectively (Fig 4B and 4C). However, when normalizing the mature hsa-miR-548ac levels to the levels of the stem-loop-containing precursor RNA, a 1.5-fold (at 24 h) and 3.4-fold (at 48 h) higher relative expression ratio was measured in mir-548ac-G-transfected cells compared with mir-548ac-A-transfected cells (Fig 4D). Although these differences were not statistically significant (Welch *t*-test *p*>0.06), the data do support a role of SNP rs1414273 in the recognition and/or processing of the primary miRNA by the Drosha-DGCR8 complex.

The real-time PCR data thus corroborated what we have seen in the eQTL analysis: The MS-associated G allele mediates a more efficient biogenesis of hsa-miR-548ac. While the effect of the genetic variant on miRNA expression is moderate, it may influence the regulation of many target genes and, in consequence, constitute an important link to the pathogenesis of MS.

### Functional analysis of microRNA-548ac

Gene expression is post-transcriptionally regulated by miRNAs via destabilization of target gene transcripts and repression of mRNA translation, although indirect mechanisms can also lead to transcriptional activation of specific genes [20,21]. We set out to identify direct target genes of hsa-miR-548ac by microarray-based screening, bioinformatic assessment, and experimental validation.

**Fig 5:**
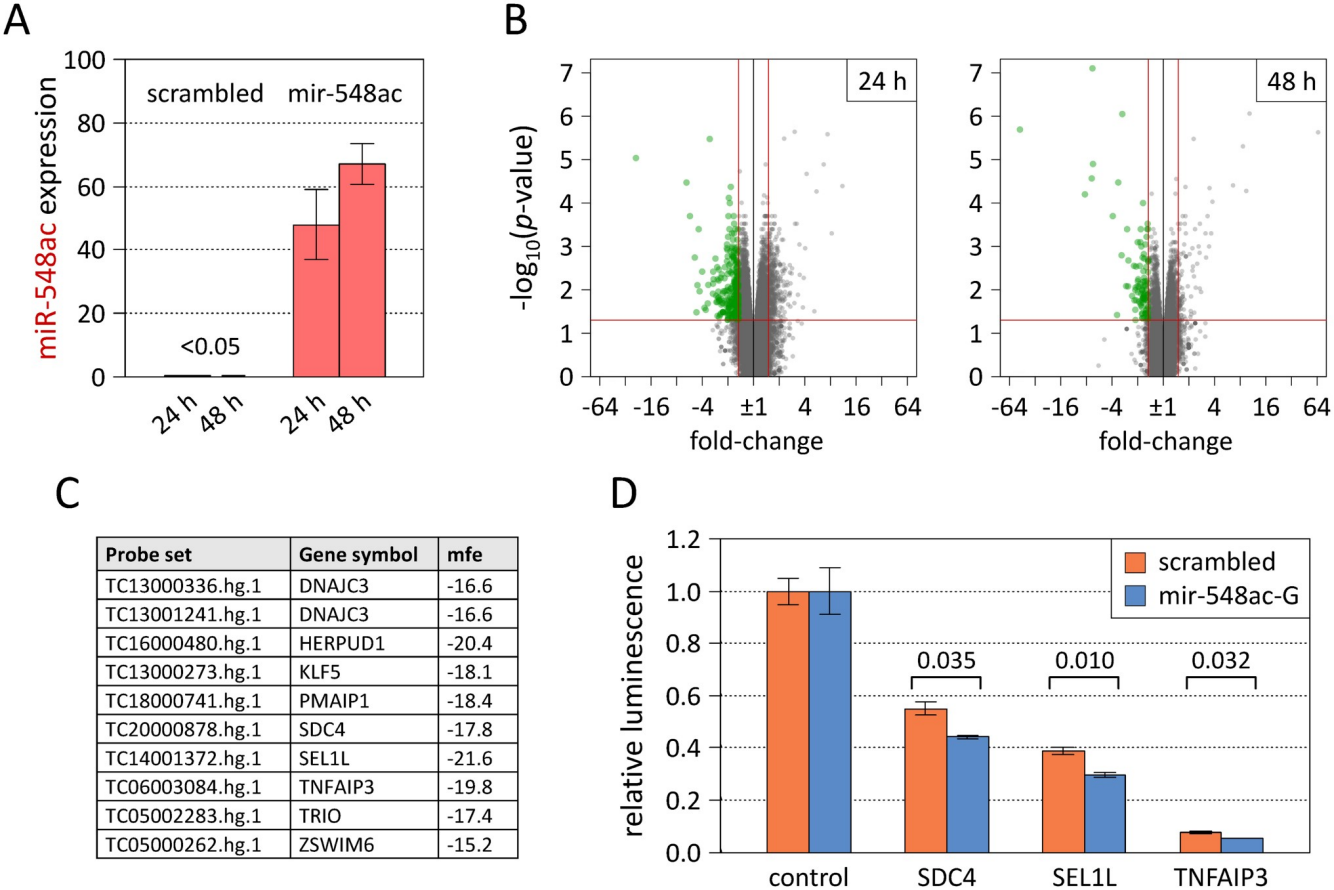
Target gene analysis of microRNA-548ac. (**A**) Expression of hsa-miR-548ac after transient transfection of HeLa cells with a precursor miRNA expression plasmid (mir-548ac-G). The level of mature miRNA molecules was determined relative to a reference miRNA (hsa-miR-191-5p) using real-time PCR based on TaqMan miRNA assays with specific stem-loop primers. Shown are means and standard errors of 2^-ΔCt^ values scaled by a factor of 1000. When using a negative control vector with scrambled sequence, hsa-miR-548ac was basically not detectable. (**B**) Volcano plots of differential gene expression in HeLa cells transfected with the mir-548ac plasmid compared to cells transfected with the control plasmid. The transcriptome profiles were obtained by HTA 2.0 microarrays, which contain 67528 probe sets for human genes. Green dots indicate probe sets with *p*-value<0.05 and fold-change≤-1.5, which corresponds to RNA levels reduced by >33.3% (n = 257 at 24 h and n = 99 at 48 h post-transfection). These significantly downregulated transcripts were bioinformatically evaluated to identify the most likely direct targets of the miRNA. (**C**) Nine protein-coding genes, which were measured by 10 probe sets, resulted as top target gene candidates (S2 Table). RNAhybrid was used to calculate the minimum free energy (mfe) for the binding of hsa-miR-548ac at the 3' untranslated region (UTR) of each mRNA [33]. (**D**) Validation assays were performed in HeLa cells with luciferase reporter plasmids containing 3' UTR sequences from SDC4, SEL1L, and TNFAIP3. GLuc/SEAP ratios are given relative to cotransfections with a 3' UTR control vector, and each bar represents the mean and standard error from 3 biological replicates. For all three miRNA 3' UTR target plasmids, the relative luciferase activities were significantly decreased 24 h after transfection with the mir-548ac plasmid compared to transfection with the plasmid expressing a scrambled sequence. Welch *t*-test *p*-values are reported above the brackets. GLuc = Gaussia luciferase, HTA = Human Transcriptome Array, PCR = polymerase chain reaction, SEAP = secreted alkaline phosphatase.

For the target screening, HeLa cells were transiently transfected with the mir-548ac-G plasmid or the control plasmid. After 24 h and 48 h, processed mature hsa-miR-548ac molecules could be measured with real-time PCR in cells transfected with the precursor miRNA expression plasmid (C_t_<34.2 for all 54 wells) but not in cells transfected with the control vector (C_t_>45 for 27 out of 36 wells). As in the former experiment (Fig 4B), the mir-548ac plasmid evoked higher levels of the miRNA at the second time point (Fig 5A). The gene expression of the cells was then profiled using high density Affymetrix Human Transcriptome Arrays (HTA) 2.0. These microarrays contain >6 million oligonucleotide probes matching to 67528 protein-coding and non-coding genes. The analysis of the data (probe set signal intensities) revealed overall 333 transcripts, which were statistically significantly downregulated by >33.3% (raw *p*-value<0.05 and fold-change≤-1.5) in mir-548ac-transfected cells as compared to negative controls (S2 Table). The expression differences were more pronounced 24 h (n = 257 transcripts) than 48 h (n = 99 transcripts) post-transfection (Fig 5B). Many of the genes are known to be expressed in blood and brain cells, and a functional analysis showed that they are significantly overrepresented in biological processes and pathways related to, e.g., "cytokine signaling in immune system", "MAPK family signaling cascades", "*de novo* posttranslational protein folding" and "response to unfolded protein" (S2 Table). Interestingly, a relatively huge fraction of transcripts (n = 50) was found to be paralogous to the mir-548 family. Their downregulation suggests that there are mutual interactions between the miRNA and various other small RNAs, which presumably evolved from the same palindromic transposable element [30]. Of the 333 potential target transcripts of hsa-miR-548ac, 6 were already listed as such in the miRTarBase catalog [31] with weak evidence from HTS data, and 35 were consistently predicted to contain a binding site for the miRNA in the 3' untranslated region (UTR) using the miRWalk 2.0 platform [32] (S2 Table). These small overlaps are not surprising due to the lack of studies on this primate-specific miRNA and the common assumption of prediction tools that binding sites should be evolutionarily conserved.

We prioritized the 333 transcripts in order to select probable direct protein-coding targets for a validation experiment. Nine genes showed consistency of downregulation, strong expression in HeLa cells, and a stable interaction of the 3' UTR with hsa-miR-548ac as calculated by RNAhybrid [33] (Fig 5C, S2 Table). Luciferase reporter assays were then performed with 3' UTR constructs for 3 of these genes: SDC4, SEL1L, and TNFAIP3. HeLa cells transfected with these constructs always produced lower relative luminescence intensities than HeLa cells transfected with a control vector containing a minimal 3' UTR. This suggests that the 3' UTRs of these genes contain binding sites for other miRNAs or other sequence features that affect mRNA stability [34]. When comparing the effect of the mir-548ac-G plasmid versus the plasmid with scrambled sequence, a significant decrease in luciferase activity was seen for SDC4 (-19.8%), SEL1L (-23.7%) as well as TNFAIP3 (-31.5%) 24 h after cotransfection (Fig 5D). As in the microarray-based screening, the regulatory effect was still present, albeit less pronounced, at the 48 h time point (Welch *t*-test *p*-value<0.05 only for SEL1L).

We could thus verify direct interactions between hsa-miR-548ac and selected candidate target genes. This establishes a functional link, where the MS-associated locus within the CD58 gene may exert its effect on disease risk by post-transcriptional modulation of gene expression via hsa-miR-548ac. The data also indicated numerous interactions between the miRNA and related non-coding transcripts, which may cause secondary effects that remain to be delineated.

### Expression of CD58 and microRNA-548ac in immune cells

Because we could detect hsa-miR-548ac molecules in all PBMC samples in the miR-eQTL analysis, we were interested in finding out which specific immune cells express this miRNA and to which extent it is coexpressed with CD58. For this purpose, we analyzed publicly available microarray and RNA-seq data for a variety of blood cell subpopulations, including T-cells, B-cells, monocytes, and natural killer (NK) cells.

**Fig 6:**
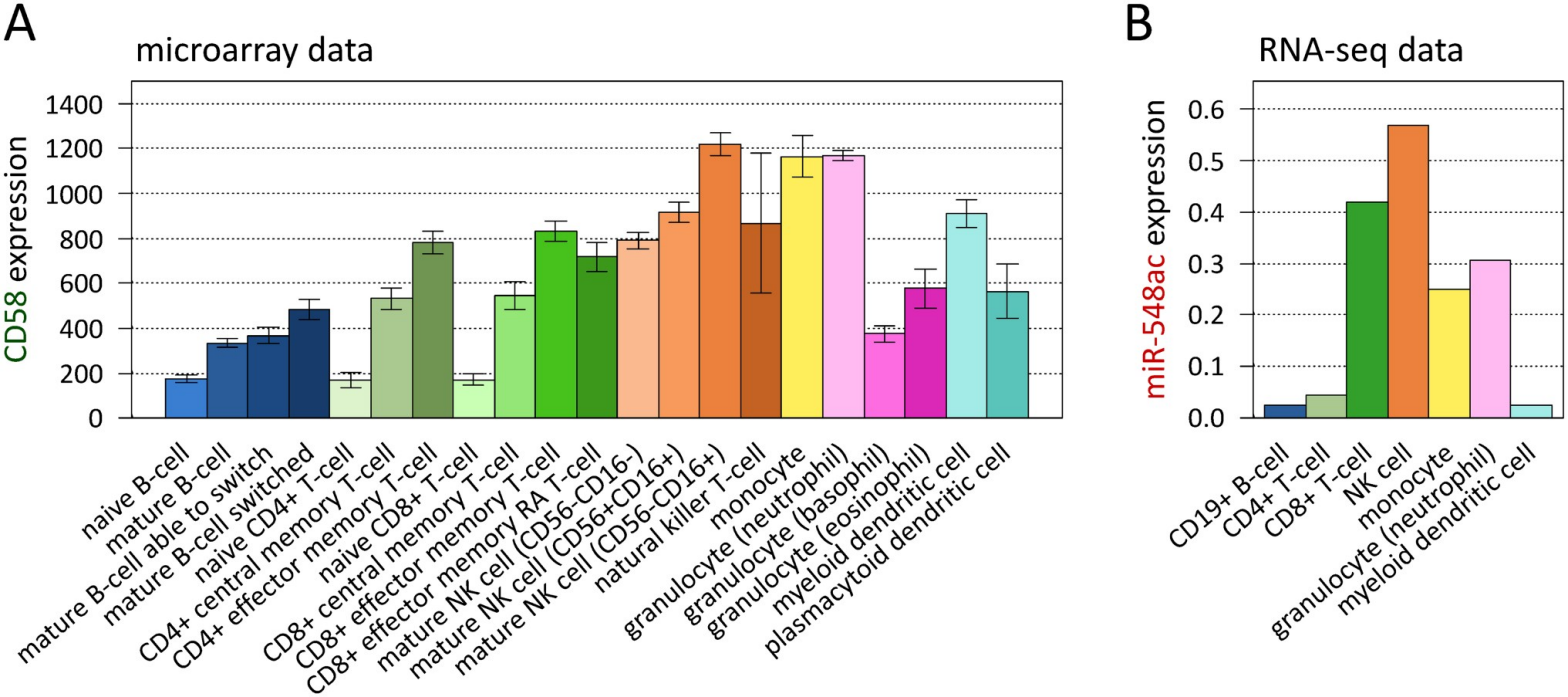
Expression of CD58 and microRNA-548ac in different immune cell populations. Cell type-specific expression was analyzed using public data on purified cell subpopulations of the blood. (**A**) This bar plot depicts the mean transcript levels of CD58 in 21 cell populations (shown in different colors) as measured by Novershtern *et al*. using microarrays [35]. The subpopulations were separated from peripheral blood samples of healthy subjects using cell surface markers. CD58 was expressed at high levels in monocytes and neutrophils as well as in subsets of T-cells, natural killer (NK) cells, and dendritic cells (DCs). The data represent probe set signal intensities in linear scale. Error bars indicate standard errors. (**B**) Visualization of the abundance of hsa-miR-548ac molecules in 7 different blood cell types. Relative expression is displayed by *z*-scores as calculated from FANTOM5 deep sequencing data [36]. The miRNA was preferentially found in CD8+ T-cells and NK cells, whereas much lower levels were noted in B-cells and myeloid DCs. This expression pattern partially reflects the expression of the host gene CD58. Divergences might be explained by uncoupling of miRNA production from the transcription of the shared primary RNA.

On the one hand, we used transcriptome profiles obtained using Affymetrix microarrays for leukocyte populations, which were separated from blood samples via cell surface markers [35]. The microarrays provided expression values for CD58 but not for hsa-miR-548ac. In these data, CD58 was expressed at high mRNA levels in NK cells, monocytes, and neutrophils, followed by effector memory T-cells and myeloid dendritic cells (DCs) (Fig 6A). On the other hand, we used the human miRNA expression atlas, which has been built based on short RNA-seq data from the Functional Annotation of Mammalian Genome 5 project (FANTOM5) [36]. This expression atlas allowed us to compare the levels of hsa-miR-548ac across 118 different cell types and tissues. The abundance of this miRNA was relatively low in 113 of the analyzed samples (*z*-score of expression <0.15). However, enriched expression (*z*-scores >0.15) was seen in CD8+ T-cells, NK cells, monocytes, and neutrophils (Fig 6B) as well as in endothelial progenitor cells, which are extremely rare in normal peripheral blood [37]. Therefore, the expression signatures of hsa-miR-548ac and its host gene CD58 were overall similar, with elevated levels in diverse immune cells, but notable differences could be ascertained in particular for CD8+ T-cells and myeloid DCs.

The observation that the miRNA and the mRNA are not necessarily proportionally produced across cell subsets suggests that cell type-specific mechanisms may modulate the processing of both molecules from the same primary transcript and/or affect their turnover. Such partial uncoupling of expression could yield a somewhat more diversified miRNA output.

## Discussion

### Characterization of an MS-associated eQTL

In this study, we investigated the regulatory effects of the MS-associated haplotype within the CD58 gene locus. The MS risk allele was associated with significantly lower CD58 mRNA levels in the HapMap microarray data and in the Geuvadis RNA-seq data. On the other hand, significantly increased levels of hsa-miR-548ac were seen in risk allele carriers in the PCR data and in the Geuvadis data. These findings suggested a genotype-dependent processing of the intronic miRNA stem-loop by Drosha-DGCR8, a mechanism which likely explains decoupling of eQTL effects for other miRNAs as well. The data exhibited two paradoxes, which may serve as examples illustrating the pitfalls of aggregating data, an issue of particular relevance in meta-analyses and multi-omics studies. Our study is the first that explicitly measured mature hsa-miR-548ac molecules in the blood of MS patients, and it is also the first that specifically aimed to identify the target genes of this miRNA. These analyses shed light on the role of this particular miRNA in MS, but also pointed out general challenges in elucidating the genetic basis of autoimmune diseases.

A relatively high proportion of MS-associated SNPs (25–50%) has been recently shown to be in strong LD with *cis*-eQTL SNPs established in blood [38–41]. In line with this, we here described an eQTL shared across multiple populations and with opposing effects on the levels of CD58 mRNA and hsa-miR-548ac in blood-derived cells, while notably the block of LD for this association does not include the gene's promoter region [16,41]. Previous studies already indicated that the haplotype, which is overrepresented in subjects with MS, is linked to moderately reduced CD58 mRNA and protein levels in LCLs [15,22,42]. We here confirmed this colocalization signal in data from more than 1000 individuals. We also showed that the eQTL effect can be obscured by differences in gene expression and genotype distribution across global populations. Moreover, we discovered a non-intuitive inverse relationship for the production of mature miRNA molecules from the same primary transcript. The further verification of this phenomenon is currently complicated by the lack of eligible miR-eQTL mapping data. In fact, the microarray- and PCR-based measurement platforms used in the majority of studies do not contain an assay for the mature form of hsa-miR-548ac, which can be explained by the fact that this miRNA is recorded in the miRBase database only since release 17 (accession MIMAT0018938) [43]. However, the rapidly increasing breadth and depth of HTS data should deliver deeper insights into genetic regulatory elements of non-coding and short RNAs (sRNA) in the near future.

We performed cell culture experiments to gain further evidence towards our hypothesis that the causal SNP of the CD58 gene locus is located in the miRNA stem-loop sequence. In these *in vitro* analyses, the ratio of mature hsa-miR-548ac miRNAs relative to precursor transcripts was up to 3.4 times higher for the MS risk allele G than for the allele A of SNP rs1414273. This difference most likely reflects an influence of this SNP at position +11 of the basal stem on the efficiency of miRNA cropping by the Drosha-DGCR8 complex. On the other hand, multiple steps affect biogenesis and turnover of miRNAs, e.g., nuclear export, Dicer-mediated processing, RNA-induced silencing complex (RISC) formation, and miRNA decay [29]. Therefore, kinetic assays may be employed to quantify the genotype-dependent velocity of Drosha cleavage more accurately [44].

### Sequence features influence microRNA processing

Others have shown that specific short sequence motifs and structural features are enriched in efficiently cleaved human primary miRNAs [20,45–47]. These findings pointed toward the base of the stem region (around position -13/+11) as a regulatory hub for miRNA processing. At the same time, a growing number of cofactors were found to enhance cleavage activity [29,48]. For instance, the prevalent CNNC motif that is also present downstream of hsa-mir-548ac (position +18 to +21) is bound by SRSF3, which is a splicing factor [45]. It is important to note that all major RNA processing activities occur cotranscriptionally at RNA polymerase II transcription sites. This includes the initial processing of miRNA stem-loops, which usually precedes intron splicing [46,49]. Introns are known to contain regulatory elements that enhance or silence exon recognition. Exonucleolytic degradation of cleaved intronic sequences thus can affect the splicing regulation of nascent transcripts [50]. As a consequence, the maturation of numerous mRNAs is expected to be influenced by Drosha activities in the nucleus [51], even if sometimes no authentic precursor miRNAs are generated for subsequent cytoplasmic processing by Dicer. While these events may provide a reason why hsa-miR-548ac miRNA levels and CD58 mRNA levels are inversely affected by the genotype, it still remains to be elucidated which precise auxiliary factors for splicing and Drosha-mediated miRNA processing may contribute to this MS-associated eQTL and whether other SNPs play a role as well. The cooperative control of the cleavage by various RNA-binding proteins allows a partial uncoupling from host gene expression and diversification of mature miRNA production from the shared primary transcript [51]. Accordingly, a transcriptome-wide analysis of chromatin-associated RNAs by Conrad *et al*. demonstrated that miRNA processing and basal transcription are independently regulated processes [46]. This is of relevance as human genetic variants can lead to structural differences in the stem-loop and in the flanking regions, thereby interfering with the recognition of primary miRNAs. In case of SNP rs1414273, there is either a Watson-Crick base pair (A-U) or a wobble base pair (G-U) at the base of the helix. Similar base substitutions have already been investigated for hsa-mir-142 [52] and hsa-mir-510 [53]. The cleavage of hsa-mir-142 precursor transcripts was also shown to be modulated in response to A-to-I editing by adenosine deaminases [52].

We identified several other SNPs with potential impact on miRNA biogenesis in the Geuvadis HTS data. This *cis*-miR-eQTL analysis revealed miRNAs encoded in the vicinity of MS-associated loci (miR-941 and miR-4664-3p) [14], miRNAs previously found to be dysregulated in MS (miR-146a-5p and miR-629-5p) [54] as well as other mir-548 family members (miR-548j-3p/-5p and miR-548l). Substantial uncoupling from host gene expression was seen for 27 mature miRNAs. However, the analysis was limited to autosomal SNPs and to miRNAs being expressed in LCLs from annotated primary transcripts. Further research is needed to obtain a better understanding of the sequence determinants and the factors that causally influence the level of miRNAs, which may provide deeper insights on the misregulation of miRNAs in disease.

### Paradoxical association patterns in eQTL data

In the eQTL analysis, we encountered a Simpson-like paradox, as the genotype effect on CD58 gene expression disappeared in the LCL data when not controlling for population substructure. Simpson's paradox refers to a sign reversal of an association between two variables, with a trend appearing in one direction for separate groups but in the opposite direction when the groups are combined [23]. We did not observe an actual reversal of the relationship between genotype and gene expression but an unexpected masking of the eQTL effect when aggregating the data of the human subpopulations. This phenomenon is of general relevance in data science and it is a reminder against simplistic reasoning, demonstrating that caution is needed when describing relations and their implications. The paradoxical element arises from incorrect assumptions and incomplete causal information. Biological and technical reasons might explain the differences in CD58 mRNA levels in LCLs between different global populations. However, it is usually not known which factors have to be included in the analysis to check whether a relationship in the data is consistent. The data can be partitioned in many ways, e.g., by gender and age of the subjects, by lab and technician involved in preparing the samples, or by means of cluster analysis. Thus, simply pooling the data of individual study cohorts can yield spurious results [55], and various elaborated statistical models have been developed to account for confounding sources of expression variation [56,57]. The literature provides examples of eQTLs that are cell type-specific, context-specific, and population-specific [38,58–63]–attributes that are not yet well represented in eQTL databases. For instance, SNP rs738289 constitutes an eQTL to two different genes from the same locus, depending on cell type: In B-cells, this SNP is associated with MGAT3 expression, whereas in monocytes, this SNP is associated with SYNGR1 expression [58]. As another example, the strength of the eQTL SNP rs285205 on MYBL2 expression is modulated by the expression of the transcription factor EBF1 [38]. The significance of a SNP might be also masked by the effects of other nearby genetic variants [64,65], which requires a conditional analysis to delineate the independent associations. Moreover, the different alleles of a SNP can be related to the expression of different RNA isoforms of the same gene (exon-level eQTLs) [38]. If such effects are not taken into consideration, a Simpson-like paradox may occur and conceal the regulatory role of a genetic variant.

We also noticed the non-transitivity of correlation [24], as hsa-miR-548ac and CD58 mRNA, which showed elevated and reduced levels in risk allele carriers, respectively, originate from the same transcript. The simulated data illustrated the paradox that discrepant directions of effect are possible in pairwise correlations between three quantitative variables. With regard to the eQTL SNP rs1414273, the relative contributions of miRNA cleavage efficiency and primary transcript expression to mature miRNA biogenesis determine the extent of uncoupling from the expression of the host gene CD58. Similar relationships can result from SNPs in bidirectional promoters or promoter-interacting regions of accessible chromatin. For instance, the alleles of SNP rs71636780 were shown to have opposite effects on the expression of ARID1A and ZDHHC18 [60]. Conversely, it is possible that two SNPs in weak LD have an independent and antagonistic regulatory influence on the level of the same transcript. Another remarkable example is the inverse miR-eQTL association that we noted for SNP rs2910164 and the two mature miRNAs from the 5' arm and the 3' arm of hsa-mir-146a. One should also keep in mind that correlations are not in general transitive when analyzing multiple types of molecular data (e.g., genetic variants, DNA methylation, chromatin state, and RNA and protein expression). Hence, proving causal links between different molecular changes is often challenging. Paradoxical conclusions might arise if the data is not supplemented with extra information of the context.

### Identification of target genes of microRNA-548ac

In our study, we aimed at further uncovering the biological implications of the MS-associated genetic locus captured by the SNP rs1414273. Whereas the host gene CD58 is known to encode a costimulatory membrane-bound protein [15,16], the functions of the intronic miRNA have not been established so far. The characterization of this particular miRNA is impeded by the fact that it belongs to a huge primate-specific family, which evolved from a transposable element [18]. As a consequence, mir-548 family members overlap in their function and probably interact with each other [30]. Their sequence similarities have complicated their discovery and their accurate annotation in the genome, and as several of these miRNAs seem to be expressed at low levels, methods with high sensitivity and specificity are needed.

For the target gene analysis of hsa-miR-548ac, we employed a precursor miRNA expression plasmid, which triggers the production of mature miRNA molecules via the endogenous miRNA biogenesis pathway. However, in comparison to the natural hsa-mir-548ac transcript, this expression construct may not adequately represent the influence of processing cofactors such as spliceosomal proteins. It is also important to note that alternative maturation might lead to different miRNA isoforms, possibly even from the 5' arm of the duplex, which may recognize different target sites. In addition, the reporter gene might carry over unprocessed miRNA stem-loops to the cytoplasm. Nevertheless, we assumed that the main regulatory effect is mediated by the canonical mature form of hsa-miR-548ac as annotated in miRBase [43] and as measured by real-time PCR. While our study benefited from a transcriptome-wide approach, we had to accept diverse limitations: (1) hsa-miR-548ac may suppress protein translation of some targets with little effect on mRNA stability, (2) fragments of degraded target RNAs may still hybridize on the microarrays, (3) feedback mechanisms may counteract the regulatory effect of the miRNA, (4) experimental conditions change over time (e.g., transient expression, miRNA turnover, and cell viability), and (5) we missed target genes not expressed in HeLa cells and non-human targets (e.g., viral genes).

The screening identified 333 potential primary and secondary target transcripts, of which 23 transcripts were filtered at both time points (24 h and 48 h). Drosha, DGCR8, and CD58 itself were not among the significantly downregulated transcripts (*p*>0.20), but there were several small non-coding RNAs with mir-548-related palindromic sequence. For 3 prioritized protein-coding genes, we could validate the binding of hsa-miR-548ac at the 3' UTR with luciferase-based assays. SDC4 is a transmembrane receptor that regulates focal adhesions [66] as well as B-cell migration and germinal center formation [67]. SEL1L guides misfolded proteins to endoplasmic reticulum-associated degradation [68] and manages a checkpoint in early B-cell development [69]. TNFAIP3 is an ubiquitin-editing enzyme involved in cytokine-mediated immune responses. Its role in various innate and adaptive immune cells has been well established [70], and multiple SNPs in the vicinity of the TNFAIP3 gene have been associated with MS (e.g., rs17780048 [14]) as well as other autoimmune diseases [70]. The gene set enrichment analysis revealed that also other putative target genes of hsa-miR-548ac are known to promote inflammatory responses and/or the folding and degradation of proteins (e.g., DNAJC3, HERPUD1, and members of the Hsp70 chaperone family). The filtering of several interferon-induced genes (e.g., IFI6 and OAS1) may correspond to previous studies indicating that mir-548 members downregulate, at least indirectly, antiviral mechanisms [71,72]. Further efforts are needed to elucidate the complex molecular network regulated by these miRNAs in order to achieve a better understanding of their role in health and disease.

### Cell type specificity of microRNA-548ac expression

To obtain further insights on hsa-miR-548ac, we had a closer look at its expression profile across cell types and tissues. The expression levels were taken from the new FANTOM5 miRNA expression atlas [36]. Compared to other data sets, this atlas provides more accurate information and includes also novel miRNAs expressed at low levels. These improvements have been achieved by advances in RNA-seq, which led to an increase in sequencing depth from nearly 1300 reads per sRNA library in the first miRNA atlas [73] to nearly 4.4 million reads per library in FANTOM5 [36]. The FANTOM5 data clearly indicated that hsa-miR-548ac is preferentially expressed in immune cells from the blood. We also found that CD58 mRNA is similarly expressed in a range of immune cells such as CD8+ T-cells, NK cells, monocytes, and neutrophils. This is a consequence of the fact that the same primary transcript is subjected to both Drosha-DGCR8 and splicing activities. However, we did expect higher levels of hsa-miR-548ac in B-cells, where it has been identified for the first time by Jima *et al*. According to their data, hsa-miR-548ac is mainly produced by lymphoma cell lines, EBV-transformed B-cells, and memory B-cells [19]. It is therefore possible that this miRNA is specifically expressed in memory B-cells but not in naive B-cells, which is the major B-cell subset in blood, especially in young people [74]. As naive and memory cells were not distinguished in FANTOM5, this remains to be investigated in further studies. Interestingly, compared to host gene expression, levels of hsa-miR-548ac were relatively high in CD8+ T-cells and relatively low in myeloid DCs. Such partial uncoupling of cell type-specific expression of CD58 mRNA and hsa-miR-548ac, if validated, might be explained by cofactors of Drosha-mediated cleavage [29,46]. Auxiliary factors for cotranscriptional splicing, RNA editing, and RNA processing may also modify the eQTL effect of SNP rs1414273 in different cell populations and at distinct stages of development. The regulation of transcription and mRNA/miRNA maturation and turnover thus deserves subsequent investigation, if possible, at single-cell resolution.

### Outlook on translating genotype to phenotype

In the past years, thousands of associations between genetic loci and human diseases have been discovered [1], with a large number of risk factors being shared across different neurological and immunological conditions [58,60,75]. However, their functional interpretation remains a challenge. Ongoing research on the causal mechanisms will deliver further insights into the pathogenesis and maintenance of such diseases and their treatment, but it has to be considered that the underlying dynamic processes constantly influence each other and thus exhibit a complex chaotic behavior [2]. One approach for addressing this issue is to employ mathematical methods that better estimate the trajectories of the biological system by integrating multiple levels of data. Fortunately, a huge diversity of large-scale molecular data sets is generated by multinational initiatives [76,77] for mapping genetic effects on gene expression, splicing, DNA methylation, histone modifications, etc. Some of these efforts also included the analysis of miRNA expression profiles from hundreds of individuals [28,78]. Still, because MS susceptibility loci may alter different processes in blood and brain, a more detailed view on specific cell types is needed to expedite the identification of causal regulatory variants. Moreover, it is important to examine combinations of genetic factors and the molecular interplay with environmental and lifestyle factors (e.g., nutrition). Studies have shown that interactions of HLA risk alleles with smoking, EBV infection, and adolescent obesity result in odds ratios for MS of >14, which is higher than the sum of the individual effects [9]. The local environment can affect disease risk phenotypes by modulating eQTL effects [63]. It is therefore difficult to disentangle beneficial and adverse gene-environment relationships. The finding that most human mRNAs are regulated by miRNAs suggests that they influence the majority of developmental processes and pathologies [20]. Intriguingly, a number of miRNAs are encoded near MS-associated tag SNPs (e.g., hsa-mir-26a-2 and hsa-mir-934) [14], and the MS risk haplotype from hsa-mir-548ac predisposes for neuromyelitis optica [79] and primary biliary cholangitis [80] as well. A better understanding of how processing, modification, and target binding of miRNAs depend on genetic variation and external stimuli is essential and may help defining biomarker constellations with large effect sizes concerning disease risk, severity, prognosis, and therapy response, which would aid in counseling patients and their relatives.

To conclude, our analysis suggests that SNP rs1414273 is possibly implicated in the development of MS. The MS-associated allele was confirmed to be linked with moderately reduced CD58 mRNA expression in blood-derived cells from more than 1000 individuals. On the contrary, HTS and real-time PCR data revealed increased levels of mature hsa-miR-548ac molecules in carriers of the risk haplotype, which we attribute to a genotype-dependent processing of the miRNA stem-loop from the first intron of the CD58 gene. We showed that hsa-miR-548ac is preferentially expressed in immune cell populations, and we found that some of its determined target genes participate in inflammation and proteostasis. Moreover, we discussed that eQTLs can be cell type-specific, context-specific, and population-specific, giving rise to paradoxical phenomena. Other disease susceptibility loci similarly contain SNPs that potentially impact the efficiency of primary miRNA cleavage and cotranscriptional splicing. As single miRNAs are able to regulate hundreds of transcripts, their aberrant expression is expected to play a critical role in various pathological conditions. The deeper investigation of disease mechanisms thus requires further studies on how genetics and environment affect cooperative miRNA-target interaction networks.

## Materials and methods

### Microarray-based eQTL analysis (HapMap cohort)

We investigated the effect of the MS-associated allele on CD58 mRNA expression using microarray data generated by the Wellcome Trust Sanger Institute for human cell lines derived from individuals that have been genetically well-characterized in the international HapMap project [25]. This analysis is very similar to the one previously performed on HapMap Phase I data [81,82] by De Jager *et al*. [15]. The main difference is that, since then, much more microarray data have been produced. Meanwhile, gene expression levels in EBV-transformed LCLs of 730 individuals from HapMap Phase II + III [25] have been measured using Illumina Human-6 v2 Expression BeadChips [26,27]. The individuals represent 8 human populations and the numbers per population are n = 112 CEU (Caucasians of northern and western European ancestry living in Utah, USA), n = 80 CHB (Han Chinese from Beijing, China), n = 82 GIH (Gujarati Indians in Houston, TX, USA), n = 82 JPT (Japanese in Tokyo, Japan), n = 83 LWK (Luhya in Webuye, Kenya), n = 45 MEX (Mexican ancestry in Los Angeles, CA, USA), n = 138 MKK (Maasai in Kinyawa, Kenya), and n = 108 YRI (Yoruba in Ibadan, Nigeria). The data in raw and normalized form are publicly available and have been downloaded from the ArrayExpress database (series accession numbers E-MTAB-198 and E-MTAB-264). With Illumina BeadChip technology, levels of RNA (but not miRNA) are quantified by hybridization to gene-specific 50mer probes. The probe identifier for CD58 is ILMN_1785268, with the corresponding oligonucleotide sequence spanning exon 2 and exon 3. Normalized signal intensity values in log2 scale were used as input for the eQTL analysis.

The genotype data were obtained from HapMap (Phase II + III) [25] and the 1000 Genomes project (Phase 3) resource [83]. SNP rs1335532 was used for distinguishing the MS risk allele (SNP rs1414273 was not covered in HapMap). Genotypes and expression data were both available for LCLs of 726 individuals. SNP information was missing for four samples (CEU: NA12274; MKK: NA21306, NA21443, and NA21649). The eQTL analysis was performed in the statistical environment R using linear models with genotypes (numerical) and populations (categorical) as independent variables (either alone or in combination) and gene expression as dependent variable. *F*-tests were calculated for all main and interaction effects based on type II sums of squares with the Anova() function of the car package for R [84]. This analysis is equivalent to SLR when using the genotype variable only. A one-way ANOVA was conducted when using the population variable only. An ANCOVA was performed for the model including both variables and a genotype-by-population interaction term. Moreover, two-sample two-tailed Welch *t*-tests were used for pairwise comparisons of genotype groups to further inspect the alleles' effect on CD58 expression. Statistical significance was defined as *p*-value<0.05.

### HTS-based eQTL analysis (Geuvadis cohort)

For further investigating the eQTL, we used data obtained by high-throughput RNA sequencing. A huge mRNA and small RNA sequencing analysis has been conducted by the Geuvadis consortium [28] on 465 LCL samples from 5 populations of the 1000 Genomes project [83]. The numbers per populations are n = 92 CEU, n = 95 FIN (Finnish in Finland), n = 96 GBR (British in England and Scotland), n = 93 TSI (Toscani in Italia), and n = 89 YRI. A subset of n = 86 CEU (93.5%) and n = 81 YRI (91.0%) individuals of the Geuvadis cohort also belongs to the HapMap cohort mentioned in the previous section. The HTS data were generated using the Illumina HiSeq 2000 platform, with paired-end 75 bp mRNA-seq and single-end 36 bp small RNA-seq. After applying quality control filters, mRNA and miRNA data were processed for 462 and 452 individuals, respectively. The Geuvadis RNA-seq data and quantification files as well as the complete genotype data are freely available in ArrayExpress (series accession numbers E-GEUV-1 and E-GEUV-2) [28]. For CD58 gene expression levels (Ensembl identifier ENSG00000116815), we downloaded the reads per kb per million mapped reads (RPKM) normalized for sequencing depth and transcript length. For hsa-miR-548ac levels, we downloaded the read counts normalized by the total number of miRNA reads. The values of replicates were averaged. Similar to the HapMap cohort analysis, we then tested whether the regulation of CD58 mRNA and hsa-miR-548ac is affected by genetic variation. The mRNA-/miR-eQTL evaluation was performed with SNP rs1414273 genotypes using *F*-tests of linear models as well as Welch *t*-tests as described for the HapMap cohort analysis.

The Geuvadis data [28] were also used for a genome-wide *cis*-miR-eQTL analysis. For all miRNAs (as of miRBase release 21) that were measured in these data together with the respective host gene (Ensembl release 90), we considered all biallelic SNPs (dbSNP build 150) located in the stem-loop or within 25 bases of flanking sequence up- and downstream of the cleavage site. SNPs in this region can lead to alterations of structural properties that are important for miRNA biogenesis, including the recognition by Drosha-DGCR8, the export to the cytoplasm, and the processing by Dicer [29,45–47]. ANCOVA were calculated for both the miRNA and the annotated primary transcript, and all SNPs with a significant genotype effect (*p*-value<0.05) on the levels of the mature miRNA were filtered. As an alternative approach, we fitted LMM that include the population variable as random-effects term (random intercept model) by minimizing the restricted maximum likelihood criterion with the lme4 R package [85] and assessed the genotype effect by type II Wald chi-square tests. Additionally, population-adjusted Pearson correlation coefficients *r* were computed for all pairwise relationships between miRNA, host gene, and SNP. These coefficients, which were based on data from 449 LCLs, were used to check for opposite directions of correlation (due to non-transitivity) and uncoupling of miRNA production from basal transcription. A difference between *r*_miRNA vs. SNP_ and *r*_host gene vs. SNP_ of >0.2 was considered as substantial uncoupling. In this case, transcription initiation and miRNA processing are suspected to be controlled in an independent and additive manner. Otherwise, the miR-eQTL association might be largely driven by other SNPs at the promoter region in the same block of LD. For all resulting gene loci, we checked for associated diseases/traits for which there are at least 10 studies in the GWAS catalog (as of 09/27/2018) [1].

### Real-time PCR-based eQTL analysis (MS cohort)

We used sensitive real-time polymerase chain reactions (PCR) for investigating the putative causal nature of the genetic risk locus within the CD58 gene in blood samples of MS patients. With written informed consent, 20 ml of peripheral venous blood was obtained from 32 MS patients (n = 27 RRMS patients in remission and n = 5 SPMS patients, n = 21 females and n = 11 males, average age of 48.3 years). The patients were diagnosed with MS according to the revised McDonald criteria [86]. Routine medical care was provided to all patients. They were treated and monitored according to the European Medicines Agency labels, following the consensus treatment guidelines and recommendations of the German Society of Neurology. The study was approved by the University of Rostock's ethics committee (permit number A 2014–0112) and carried out in line with the Declaration of Helsinki. DNA was purified from the blood samples using the PAXgene Blood DNA Kit (Qiagen). PBMC were separated using a Ficoll gradient (Histopaque-1077, Sigma-Aldrich) and stored in mirVana lysis buffer at -20 °C. Total RNA enriched with small RNAs was isolated using the mirVana miRNA Isolation Kit (Thermo Fisher Scientific).

Genotyping was done by PCR with allele-specific TaqMan probes for SNP rs1414273, which is located at the base of the hsa-mir-548ac stem-loop sequence [16]. The Custom TaqMan Assay Design Tool (Thermo Fisher Scientific) was used to construct the probes and primers (forward primer: 5'-ACCTGGTATTAAAAAGTGGAACATAAAATCTCT-3', reverse primer: 5'-ATGGCAAAAACCGGCAATTACTTT-3', VIC dye-labeled TaqMan minor groove binder (MGB) probe: 5'-TGCACTAACCTAATAGTTAC-3', FAM dye-labeled TaqMan MGB probe: 5'-TGCACTAACCTAGTAGTTAC-3'). PCR amplification was carried out following manufacturer's instructions in a 7900HT Fast Real-Time PCR System (Applied Biosystems). Endpoint analysis was used to determine the genotypes of the individual patients by allelic discrimination.

Quantitation of transcript levels and miRNA expression was performed with TaqMan single-tube assays from Thermo Fisher Scientific. We measured CD58 (assay identifier Hs01560660_m1, amplicon spanning exon 1 and exon 2) and hsa-miR-548ac (464325_mat) as well as GAPDH (Hs99999905_m1) and hsa-miR-191-5p (002299) [87] as reference gene and miRNA, respectively. From each sample, 400 ng of total RNA was reverse transcribed with random primers for mRNA species (High-Capacity cDNA Reverse Transcription Kit), and 10 ng of total RNA was reverse transcribed with specific primers provided with each TaqMan miRNA assay to convert mature miRNAs to cDNA (TaqMan MicroRNA Reverse Transcription Kit, Thermo Fisher Scientific). The quantitative real-time PCR was then run in triplicates with the predesigned primers and TaqMan probes according to the manufacturer's protocols with 45 cycles in the 7900HT instrument (Applied Biosystems). Threshold cycle (C_t_) values were computed using SDS 2.3 and RQ Manager 1.2 software (Applied Biosystems) (S3 Table).

For mRNA and miRNA data preprocessing, we calculated the mean C_t_ value of each triplicate, normalized the means to those of reference gene and miRNA (ΔC_t_ method), converted the values to the linear scale using the equation 2^-ΔCt^, and scaled the result by a factor of 1000 for convenience. Afterwards, SLR was used for the eQTL analysis based on CD58 gene expression, hsa-miR-548ac levels, and the number of SNP rs1414273 risk alleles per patient. Welch *t*-tests at the 0.05 level of significance were calculated for pairwise comparisons of genotype groups.

### Simulation of eQTL paradoxes

We simulated genotype distributions and gene expression data to illustrate that counter-intuitive results may be obtained in eQTL analyses. The first scenario is posed by Simpson's paradox and the second by the non-transitivity paradox of correlation. Simpson's paradox (also called amalgamation paradox) refers to phenomena, in which a trend appears in different groups of data but reverses when these groups are combined [23]. Formally, a relationship between two variables A and B is seen in one direction when partitioning the data by variable C, but the opposite direction is seen when variable C is ignored. According to the classical definition, the variables A, B, and C are categorical, but the paradox applies to quantitative data as well. Moreover, while Simpson's paradox denotes an actual reversal in the relationship when not controlling for the confounding variable C [23], we here consider associations that disappear rather than reverse signs upon data aggregation. This more general statistical problem has been referred to as Simpson-like paradox [88]. In eQTL studies, gene expression levels (variable A) are compared between genotypes (variable B) in data obtained from individuals that can be grouped by demographic information, such as population structure (variable C). Simpson-like paradox then happens if two events occur together: (1) the number of individuals per genotype × subpopulation group are very different and (2) the population has a large effect on the measured gene expression. An exemplary data set was simulated in R with 60 individuals for each genotype, with the major allele in one population being the minor allele in the other population, and with a continuous genotype-dependent trend in the gene expression values (means of 15, 10, and 5 for the first population and means of 30, 25, and 20 for the second population). The data were then analyzed using linear models to demonstrate that the eQTL association disappears when examining the data without accounting for subpopulations.

A second exemplary data set was used to show that correlation is not transitive [24]. Due to this, when considering a chain of correlation where A correlates with B and B correlates with C, it does not necessarily follow that A correlates with C. This peculiarity is often regarded as paradoxical, but it is basically just a reminder that correlation is not causation, because the three bivariate relationships may have different underlying reasons. We simulated expression data of two RNAs (variables A and B) for 60 individuals equally distributed among the three possible genotypes of a biallelic SNP (variable C). The variables for the two RNAs are quantitative and we also treated the variable for the SNP as quantitative (representing the number of a certain allele). We supposed the RNAs to be positively correlated in expression with some random variation in the data. Additionally, we introduced an inverse dependency on the genotype for the levels of both RNAs. The eQTL effect was then assessed by SLR, which is equivalent to calculating the *p*-value for Pearson's product-moment correlation.

### Expression of microRNA-548ac in HeLa cells to study the SNP effect

We performed cell culture experiments to analyze whether the genotype of SNP rs1414273 is linked to mature miRNA levels. For this purpose, a miExpress precursor miRNA expression plasmid was purchased from GeneCopoeia (HmiR1085-MR04) and amplified in *E*. *coli*. This plasmid contains a 315 bp long DNA fragment from the first intron of the CD58 gene that comprises the miRNA stem-loop sequence as well as flanking regions. This fragment is coexpressed with a reporter gene coding for enhanced green fluorescent protein as a combined transcript. The expression of this construct is driven by a cytomegalovirus promoter (vector pEZX-MR04).

A second plasmid was derived that only differs from the ordered plasmid at the position of SNP rs1414273. The ordered plasmid encodes the G allele of this SNP at the base of the hsa-mir-548ac stem-loop (mir-548ac-G). We generated a G to A base substitution using the QuikChange Lightning Site-Directed Mutagenesis Kit (Agilent Technologies) and two oligonucleotide primers that contain the desired mutation and anneal to the same sequence on opposite strands of the plasmid (Life Technologies, forward primer: 5'-TTTTGCACTAACCTAATAGTTACTACAAAAA-3', reverse primer: 5'-TTTTTGTAGTAACTATTAGGTTAGTGCAAAA-3'). The mutagenesis was verified by bidirectional sequencing using a GenomeLab GeXP Genetic Analysis System (Beckman Coulter). The sequencing primers were designed as suggested in the vector data sheet from GeneCopoeia and synthesized by Life Technologies (forward primer: 5'-CCGACAACCACTACCTGA-3', reverse primer: 5'-ATTGTGGATGAATACTGCC-3'). Visual inspection of the obtained chromatograms was done with the Chromas 2.6.4 software (Technelysium).

For the comparative analysis, HeLa cells were transiently transfected with either precursor miRNA expression plasmid (mir-548ac-G and mir-548ac-A) using the FuGENE HD transfection reagent (Promega). A vector with a scrambled sequence (CmiR0001-MR04, GeneCopoeia) served as negative control. Total RNA was isolated 24 h and 48 h after transfection with the miRNeasy Mini Kit (Qiagen) and an on-column DNase digestion step. All these experimental procedures were done in duplicates, which resulted in 12 different RNA samples. Reverse transcription and quantitative real-time PCR were then conducted basically as described in section "Real-time PCR-based eQTL analysis (MS cohort)". In brief, 400 ng and 10 ng of total RNA from each sample were reverse transcribed for the analysis of mRNA and miRNA, respectively. TaqMan single-tube assays from Thermo Fisher Scientific were employed for the detection of mature hsa-miR-548ac molecules as well as their precursor transcripts. The custom assay for the primary RNA was designed with the Primer Express 3.0 software (Applied Biosystems) so that the PCR amplicon contains the entire miRNA stem-loop sequence (forward primer: 5'-GGCTAAAGAAGTGCTCTCAAATAGAAG-3', reverse primer: 5'-GACCTGGTATTAAAAAGTGGAACATAAA-3', FAM dye-labeled TaqMan MGB probe: 5'-TTTCATTTCGACATGTATTAGG-3'). Additionally, GAPDH and the reference miRNA hsa-miR-191-5p were measured for normalization [87]. The real-time PCR was performed in triplicates with 45 cycles in a 7900HT Fast Real-Time PCR System (Applied Biosystems). The obtained C_t_ values are provided in S3 Table.

The relative abundance of hsa-mir-548ac precursor RNAs and mature miRNAs was assessed based on the ΔC_t_ method. Accordingly, median C_t_ values per triplicate were normalized to those of reference gene and miRNA, respectively (ΔC_t_), and then transformed to linear scale (2^-ΔCt^). To compare the two SNP rs1414273 alleles with regard to hsa-miR-548ac expression, the mature miRNA levels were normalized to the precursor levels using the 2^-ΔΔCt^ method, with $\Delta \Delta {\text{C}}_{\text{t}}=({\text{C}}_{{\text{t}}_{\text{miR}-548\text{ac}}}-{\;\text{C}}_{{\text{t}}_{\text{miR}-191-5\text{p}}})-({\text{C}}_{{\text{t}}_{\text{precursor}\;\text{mir}-548\text{ac}}}-{\;\text{C}}_{{\text{t}}_{\text{GAPDH}}})$. Finally, the values in linear scale were scaled so that the data for the A allele and the 24 h time point are equal to 1, respectively. Bar plots were created for displaying the resulting expression ratios.

### Screening for potential target genes of microRNA-548ac

For the initial screening, we conducted a transcriptome profiling analysis of HeLa cell cultures. The cells were transiently transfected with a non-viral precursor miRNA expression plasmid (mir-548ac-G) or the negative control with scrambled sequence, and total RNA from these cells was prepared for real-time PCR analysis to confirm *in vitro* expression of mature hsa-miR-548ac molecules essentially as described in the previous section. The RNA samples were isolated 24 h and 48 h after transfection of the mir-548ac plasmid (n = 9 replicates) or the control plasmid (n = 6 replicates). The quantitative real-time PCR was then run with 45 cycles, and if the signal did not pass the threshold, the data were set to C_t_ = 45. The resulting C_t_ values (S3 Table) were averaged over triplicates, normalized to the reference miRNA hsa-miR-191-5p [87], transformed to linear scale, and scaled. A subset of 10 RNA samples was used for the microarray gene expression analysis, with triplicates and duplicates from both time points for mir-548ac-transfected cells and negative controls, respectively. To this end, 210 ng of total RNA from each sample was used as starting material for the Affymetrix GeneChip Whole Transcript Sense Target Labeling Assay protocol, which allows to generate amplified, fragmented, and biotinylated single-stranded sense strand DNA from the entire expressed genome. The hybridization on high-resolution HTA 2.0 microarrays (Affymetrix) was then carried out for 16 h at 45 °C in a GeneChip Hybridization Oven 645 (Affymetrix) following the manufacturer's protocol. After washing and staining in the Affymetrix Fluidics Station 450, the microarrays were scanned using a GeneChip Scanner 3000 7G (Affymetrix). The scanned images were imported to the Affymetrix GeneChip Command Console version 4.0 to extract the signal intensities for the >6 million 25mer oligonucleotide probes per array. The data were further processed using the Transcriptome Analysis Console (TAC) version 4.0 (Affymetrix) with the signal space transformation robust multi-array average (SST-RMA) algorithm for background adjustment, quantile normalization, gene-level probe set summarization, and log2 transformation. This resulted in intensity values for 44699 probe sets for protein-coding genes and 22829 probe sets for non-coding genes, including many miRNA precursors. The raw and processed microarray data are publicly available in the GEO database (accession number GSE120769).

Binding of RISC-miRNA complexes to complementary sites primarily mediates RNA decay [20]. We thus examined the microarray data for significantly downregulated transcripts in mir-548ac-G-transfected HeLa cells. The analysis of differential expression was performed according to the default workflow in TAC 4.0, which is based on the limma method with eBayes correction [89]. Fold-changes were calculated in linear scale from robust means per sample group, while in case of downregulation, the ratio has been inverted and multiplied by -1. For both the 24 h and the 48 h time point, we filtered probe sets with fold-change≤-1.5 at the nominal significance level of α = 5%.

A series of database-driven analyses was done with the obtained combined list of potential target genes of hsa-miR-548ac. First, we used the web tool Enrichr [90] to determine Gene Ontology biological process categories [91] and Reactome cell signaling pathways [92] with a combined gene set enrichment score >20. Second, we studied the genes' expression in cell types of the blood and brain based on the microarray data from Novershtern *et al*. [35] (GEO series GSE24759) and the RNA-seq data from Darmanis *et al*. [93] (downloaded via http://celltypes.org/brain/ [94]) and by demanding an RNA level of >100. Third, we checked whether the filtered probe sets interrogate transcripts, which are sequence-related to the mir-548 family. For this purpose, the corresponding genetic sequences were fetched from the reference genome (GRCh37 assembly) and tested for similarity with human mir-548 stem-loop sequences using the search tool in miRBase release 21 [43] with E-value<10 and score>100 as cutoffs. Fourth, we evaluated whether the genes were also predicted to be regulated by hsa-miR-548ac in the miRWalk 2.0 interaction information retrieval system [32]. We requested that at least 6 out of the 12 different algorithms provided by the web-interface consistently predict the binding of the miRNA at the respective 3' UTRs. Fifth, we used miRTarBase release 6.1 [31] to determine the overlap with experimentally determined miRNA targets collected from the literature.

### Validation of direct target genes by luciferase reporter assays

Because some gene expression changes observed in the screening experiment may result from secondary effects, we prioritized the genes in order to select the most likely direct target transcripts of hsa-miR-548ac for validation. On the one hand, we determined the subsets of genes, which were nominally downregulated at both time points, that is 24 h and 48 h post-transfection with the mir-548ac plasmid, and which showed robust expression in HeLa cells, with an average log2 signal >10 for either group of samples transfected with the negative control plasmid. On the other hand, we used the RNAhybrid program (version 2.2) [33] for assessing evidence of direct miRNA-target interactions *in silico*. RNAhybrid determines the most favorable hybridization of two RNAs. As input, we used the 3' UTR sequences of the downregulated protein-coding transcripts, which were gathered via the BioMart portal of the Ensembl database (release 89) [95,96]. Moreover, we opted for a maximum free energy of -15 and a helix constraint for the seed region of the miRNA (position 2 to 7). The result of this analysis revealed a subset of genes with at least one predicted 3' UTR binding site for hsa-miR-548ac.

Luciferase reporter assays were conducted for 3 of the prioritized target genes. For this purpose, miTarget miRNA 3' UTR target plasmids (vector pEZX-MT05) were purchased from GeneCopoeia for SDC4 (HmiT016661-MT05), SEL1L (HmiT016743-MT05), TNFAIP3 (HmiT061984-MT05), and a negative control (CmiT000001-MT05). These constructs enable the *in vitro* expression of the respective 3' UTR sequences as insert downstream of a reporter gene (Gaussia luciferase, GLuc), and they contain a second constitutively expressed reporter (secreted alkaline phosphatase, SEAP) as an internal control. They were cotransfected with the mir-548ac-G plasmid or the scrambled sequence expression plasmid in HeLa cells in 3 biological replicates. Following transfection for 24 h and 48 h, supernatants were collected, and GLuc and SEAP activities were measured twice using the Secrete-Pair Dual Luminescence Assay Kit (GeneCopoeia) and a GloMax-Multi Detection System (Promega) according to the manufacturers' recommendations (S3 Table). Afterwards, ratios of the average luminescence catalyzed by GLuc and SEAP were calculated, normalized to those resulting for cotransfections with the 3' UTR control vector, and compared using Welch *t*-tests.

### Cell type-specific expression in blood

We analyzed microarray and RNA-seq data to compare the levels of CD58 mRNA and hsa-miR-548ac in different immune cell populations circulating in the blood. The microarray data set was generated by Novershtern *et al*. using Affymetrix Human Genome U133 GeneChips [35]. These data provided gene expression profiles for purified subpopulations of B-cells, T-cells, NK cells, monocytes, granulocytes, and DCs from the blood of healthy volunteers. We downloaded the RMA-normalized data from the GEO repository (accession number GSE24759) and extracted the signal intensities for CD58 as summarized on the basis of 11 different 25mer oligonucleotide probes (probe set identifier 205173_x_at).

The cell type-specific abundance of mature hsa-miR-548ac molecules was evaluated using the expression atlas of human miRNAs, which has been recently created as part of the FANTOM5 project [36]. In FANTOM5, deep sequencing of sRNA libraries from a large collection of human primary cells, cell lines, and tissues has been performed using an Illumina HiSeq 2000 sequencer. The number of sRNA tags to each miRNA locus were counted, normalized to counts per million, averaged across sample donors, and converted to *z*-scores. The *z*-scores for hsa-miR-548ac were taken from the mature miRNA expression table provided at the FANTOM5 web interface. Bar plots were used for displaying the levels of hsa-miR-548ac and CD58 mRNA in leukocyte populations.

## Supporting information

S1 Table Global analysis of eQTL paradoxes for human miRNAs. This table lists *cis*-miR-eQTLs detected in high-throughput sequencing data of the Geuvadis consortium [28]. Genetic variants underlying these eQTL associations might modulate transcriptional regulation, splicing efficiency, and/or enzymatic cleavage activity of Drosha and Dicer. Identifiers and chromosome positions (in ascending order) are given for all miRNAs, host genes, and single-nucleotide polymorphisms (SNPs). Diseases/traits potentially related to the genes were retrieved from the GWAS catalog [1]. The SNP alleles are specified for the forward strand (fwd) of the reference genome (GRCh38 assembly). Moreover, the position of each SNP within the miRNA stem-loop sequence (- = upstream, + = downstream, P = precursor molecule) and the effect on mature miRNA expression are indicated. *F*-test *p*-values of the linear models as well as Pearson correlation coefficients were compared for detecting instances of the Simpson-like paradox and the non-transitivity paradox. Additionally, Wald chi-square test *p*-values are provided for the genotype effect estimated for linear mixed-effects models (LMM) that incorporate the heterogeneity across human populations via a random-effects term. ANCOVA = analysis of covariance, ANOVA = analysis of variance, eQTL = expression quantitative trait locus, GWAS = genome-wide association study, HGNC = Human Genome Organisation Gene Nomenclature Committee, ID = identifier, SLR = simple linear regression.
(XLS)

S2 Table Detailed information for the 333 candidate target transcripts of microRNA-548ac that were identified by microarray-based screening. Affymetrix Human Transcriptome Arrays (HTA) 2.0 were employed for measuring the gene expression profiles of HeLa cells, which were transiently transfected with either a plasmid for *in vitro* expression of the miRNA or a negative control plasmid with scrambled sequence. Transcripts showing reduced levels after 24 h or after 48 h in mir-548ac-G-transfected cells (with *p*-value<0.05 and fold-change≤-1.5) were considered as being regulated by hsa-miR-548ac directly or indirectly. This table provides the probe set identifiers, official gene symbols (if available), Entrez Gene database identifiers (ID), the number of 25mer oligonucleotide probes per probe set, robust means (Tukey's biweight of log2 signals) for each group of samples (at least duplicates) as well as the results of the differential expression analysis. The fold-changes are given for the data on linear scale, and they have a negative sign if the levels were higher in the negative control samples. Additional columns define the criteria, which were used to prioritize a subset of most likely target genes for the validation experiment with luciferase reporter assays. Moreover, we provide information on sequence similarities with mir-548 family members and whether there is already evidence of an interaction in miRNA databases [31,32]. The rightmost columns specify whether the genes are expressed in different cell types of the blood and brain (- = not measured). The results of the gene set enrichment analysis are presented on the second sheet. See the "Materials and methods" section for more details. DC = dendritic cell, NK cell = natural killer cell, NKT cell = natural killer T-cell, OPC = oligodendrocyte precursor cell.
(XLS)

S3 Table The full data sets underlying the reported findings from quantitative real-time PCR analyses and luciferase reporter assays. The levels of CD58 mRNA, hsa-miR-548ac, and reference gene/miRNA were measured in PBMC samples of 32 MS patients in triplicates. Moreover, hsa-miR-548ac, the stem-loop-containing precursor RNA as well as a reference gene/miRNA were measured in HeLa cells 24 h and 48 h after transfection with 3 different plasmids (mir-548ac-G, mir-548ac-A, and negative control) using biological duplicates and technical triplicates. Mature hsa-miR-548ac molecules were also quantified in triplicates relative to a reference miRNA in HeLa cells at 24 h and 48 h post-transfection with the mir-548ac-G plasmid (n = 9 replicates) or the control plasmid (n = 6 replicates). For the target gene validation experiment, these two plasmids were cotransfected with 4 different luciferase reporter plasmids in 3 biological replicates, and GLuc and SEAP activities were then measured twice after 24 h and 48 h. GLuc = Gaussia luciferase, MS = multiple sclerosis, PBMC = peripheral blood mononuclear cells, PCR = polymerase chain reaction, SEAP = secreted alkaline phosphatase.
(XLS)
